# A Novel Orthotopic Implantation Technique for Osteosarcoma Produces Spontaneous Metastases and Illustrates Dose-Dependent Efficacy of B7-H3-CAR T Cells

**DOI:** 10.3389/fimmu.2021.691741

**Published:** 2021-06-15

**Authors:** Lindsay Jones Talbot, Ashley Chabot, Amy Funk, Phuong Nguyen, Jessica Wagner, Aaron Ross, Heather Tillman, Andrew Davidoff, Stephen Gottschalk, Christopher DeRenzo

**Affiliations:** ^1^ Department of Surgery, St. Jude Children’s Research Hospital, Memphis, TN, United States; ^2^ Department of Veterinary Medicine, St. Jude Children’s Research Hospital, Memphis, TN, United States; ^3^ Department of Bone Marrow Transplant and Cellular Therapy, St. Jude Children’s Research Hospital, Memphis, TN, United States; ^4^ University of Tennessee Health Sciences School of Medicine, Memphis, TN, United States; ^5^ Department of Pathology, St. Jude Children’s Research Hospital, Memphis, TN, United States

**Keywords:** osteosarcoma, orthotopic, model, CAR, T cell therapy, B7-H3

## Abstract

The outcome for metastatic pediatric osteosarcoma (OS) remains poor. Thus, there is an urgent need to develop novel therapies, and immunotherapy with CAR T cells has the potential to meet this challenge. However, there is a lack of preclinical models that mimic salient features of human disease including reliable development of metastatic disease post orthotopic OS cell injection. To overcome this roadblock, and also enable real-time imaging of metastatic disease, we took advantage of LM7 OS cells expressing firefly luciferase (LM7.ffLuc). LM7.ffLuc were implanted in a collagen mesh into the tibia of mice, and mice reliably developed orthotopic tumors and lung metastases as judged by bioluminescence imaging and histopathological analysis. Intratibial implantation also enabled surgical removal by lower leg amputation and monitoring for metastases development post-surgery. We then used this model to evaluate the antitumor activity of CAR T cells targeting B7-H3, an antigen that is expressed in a broad range of solid tumors including OS. B7-H3-CAR T cells had potent antitumor activity in a dose-dependent manner and inhibited the development of pulmonary metastases resulting in a significant survival advantage. In contrast T cells expressing an inactive B7-H3-CAR had no antitumor activity. Using unmodified LM7 cells also enabled us to demonstrate that B7-H3-CAR T cells traffic to orthotopic tumor sites. Hence, we have developed an orthotopic, spontaneously metastasizing OS model. This model may improve our ability not only to predict the safety and efficacy of current and next generation CAR T cell therapies but also other treatment modalities for metastatic OS.

## Introduction

Osteosarcoma (OS) is the most common tumor of bone in children and adolescents, and the third most common solid tumor encountered in this age group. While great success has been achieved with local control modalities over the last thirty years, resulting in a survival rate from primary OS of approximately 60 – 75% depending on histologic response, the treatment of recurrent and metastatic disease remains less effective (1–3). This is in part due to a lack of highly relevant pre-clinical models (4), prohibiting the realistic screening and modeling of therapeutic approaches.

Current preclinical orthotopic models of OS have varying success in reproducing clinically relevant metastatic processes, which include escape from the primary tumor, navigation of stromal interactions, bloodstream entry, vascular arrest, extravasation, and establishment of a pro-tumorigenic microenvironment in the metastatic niche (5–7). Limitations of current models include a low rate of systemic or pulmonary metastasis in subcutaneous and fragment-implantation models, and inadvertent seeding of the pulmonary vasculature with tumor cells after marrow-cavity orthotopic injections. These models, while contributing substantially to the preclinical literature in OS, either do not reliably recapitulate the clinical metastatic process or have lower rates of metastasis that hamper feasibility of use.

Here, we developed a novel spontaneously metastasizing orthotopic OS model and explored its utility to evaluate the efficacy of chimeric antigen receptor (CAR) T cells. CAR T cell therapy has shown considerable preclinical promise in pediatric sarcoma models (8–12). However, while early clinical trials have demonstrated feasibility and safety, clinical responses have thus far been disappointing, highlighting the need to ensure that preclinical models mimic the clinical setting while maintaining feasibility (12–14).

We show that collagen-tumor cell scaffolds surgically implanted into the tibia of mice reliably produced local and systemic metastatic OS. Likewise, we demonstrate that CAR T cells targeting B7-H3, a tumor antigen that is expressed in a high percent of OS (15–17), have antitumor activity in a dose dependent fashion against primary and metastatic OS. Thus, the described model should be highly relevant for the preclinical evaluation and optimization of cell-based immunotherapies.

## Materials and Methods

### Cell Lines

The OS cell line LM7, a derivative of SaOS-2, was kindly provided by Dr. Eugenie Kleinerman (MD Anderson Cancer Center, Houston, TX, USA) (18). LM7 cells expressing green fluorescent protein (GFP) and firefly luciferase (ffluc) (LM7.ffluc) previously generated in our laboratory were used for all experiments (15). LM7 was grown in DMEM (GE Healthcare, Marlborough, MA, USA) supplemented with 10% fetal bovine serum (GE Healthcare) and 1% Glutamax (Thermo Fisher Scientific, Waltham, MA, USA). Cell subculture was performed by detaching adherent cells using 0.05% trypsin-EDTA (Thermo Fisher Scientific). BV173 leukemic cells (German Collection of Microorganisms and Cell Cultures, Braunschweig, Germany) were cultured in RPMI (GE Healthcare) supplemented with 10% fetal bovine serum and 1% Glutamax. All cells were maintained at 37°C in 5% CO_2_. Cell lines were authenticated by STR profiling and checked routinely while in culture for mycoplasma using the MycoAlert mycoplasma detection kit (Lonza).

### Generation of B7-H3-CAR and Control Lentiviral Vectors

The lentiviral vectors encoding B7-H3.CD8α.CD28ζ and B7-H3.CD8α.Δ (nonsignaling control) CARs were previously described (15). VSVG-pseudotyped lentiviral particles were produced by St. Jude Children’s Research Hospital Vector Core as previously described (19).

### Generation of B7-H3-CAR and Control CAR T Cells

Human peripheral blood mononuclear cells (PBMCs) were obtained from whole blood of healthy donors under an Institutional Review Board (IRB)-approved protocol at St. Jude Children’s Research Hospital after informed written consent was obtained in accordance with the Declaration of Helsinki. The generation of CAR T cells was previously described (15). Briefly PBMCs were isolated by Lymphoprep (Abbott Laboratories, Abbott Park, IL, USA) gradient centrifugation. On day -1, CD4+ and CD8+ T cells were enriched from PBMCs by immunomagnetic separation using CD4 and CD8 microbeads (Miltenyi, Germany), an LS column (Miltenyi), and a MidiMACS separator (Miltenyi). Enriched T cells were resuspended at 1 x 10^6^ cells/mL in RPMI 1640 (GE Healthcare) supplemented with 10% FBS (GE Healthcare), 1% GlutaMAX (Thermo Fisher Scientific), and cytokines IL7 and IL15 (10 ng/mL each) (Biological Resources Branch, National Cancer Institute, Frederick, MD, USA, and PeproTech, Rocky Hill, NJ, USA) and stimulated overnight on 24-well non-tissue-culture treated plates that were precoated with CD3 and CD28 antibodies (Miltenyi). Transduction was performed on day 0 by adding LV particles at an MOI of 50 TU/cell and protamine sulfate at 4 μg/mL. On day 3, T cells were transferred into new 24-well tissue culture treated plates and subsequently expanded with IL7 and IL15 (10 ng/mL each). All experiments were performed 7 – 14 days post-transduction. Biological replicates were performed using PBMCs from different healthy donors.

### Flow Cytometry

A FACSCanto II (BD Biosciences) instrument was used to acquire flow cytometry data, which was analyzed using FlowJo v10.7 (BD Biosciences). For surface staining, samples were washed with and stained in PBS (Lonza) with 1% FBS (GE Healthcare). For all experiments, matched isotypes or known negatives (e.g. nontransduced T cells or B7-H3-negative cell lines) served as gating controls. CAR detection was performed using F(ab’)_2_ fragment-specific antibody (polyclonal, Jackson ImmunoResearch, West Grove, PA, USA). T cells were stained with fluorochrome-conjugated antibodies using combinations of the following markers: CD4 (clone SK3, BD Biosciences), CD8 (clone SK1, BD Biosciences), CCR7 (clone G043H7, BioLegend, San Diego, CA, USA), and CD45RO (clone UCHL1, BD Biosciences). LM7 and the negative control leukemia cell line BV173 were evaluated for expression of B7-H3 using B7-H3 antibody (clone 7-517, BD Biosciences, or clone FM276, Miltenyi). Cells were additionally stained with DAPI (BD Biosciences) to gate for live cells.

### Analysis of Cytokine Production

T cells were cultured alone or with LM7 tumor cells at a 1:1 effector to target ratio without the provision of exogenous cytokines. Approximately 24 hours after coculture initiation, supernatant was collected and frozen for later analysis. Production of IFNγ and IL2 was measured using a quantitative ELISA per the manufacturer’s instructions (R&D Systems, Minneapolis, MN, USA).

### Antigen-Stimulated Expansion Assay

T cells were cultured alone or with LM7 tumor cells at a 1:1 effector to target ratio without the provision of exogenous cytokines. Approximately 72 hours after coculture initiation, T cells were removed from coculture and replated in fresh complete media. Following 4 additional days of culture, T cells were counted and fold change from baseline was calculated.

### Cytotoxicity Assay

The xCELLigence real-time cell analyzer (RTCA) MP instrument (Agilent Technologies, Santa Clara, CA, USA) was used to assess CAR T cell cytotoxicity. All assays were performed in triplicate and without the addition of exogenous cytokines. First, 30,000 LM7 cells in complete RPMI were added to each well of a 96-well E-plate (Agilent). After LM7 cells adhered to the E-plate for approximately 24 hours and reached a cell index (relative cell impedance) plateau, 150,000 T cells in complete RPMI were added. LM7 cells alone served as a tumor only control and LM7 cells in DMSO served as a full lysis control. The cell index was monitored every 15 minutes for 24 hours and normalized to the maximum cell index value immediately prior to T cell plating. Percent cytotoxicity was calculated using the RTCA Software Pro immunotherapy module (Agilent) (20).

### Orthotopic Modeling Technique

#### Mice

Eight-week-old, female, NSG (NOD.*Cg-PrkdcscidIl2rgtm1Wjl*/SzJ) mice were purchased from The Jackson Laboratory (Bar Harbor, ME, USA), with all animal procedures reviewed and approved by the St. Jude Children’s Research Hospital Institutional Animal Care and Use Committee. Mice underwent orthotopic tibial implantation as described below and were followed by weekly bioluminescence. They underwent hindlimb amputation when they reached a humane endpoint that included lameness, large tumor burden interfering with the animal’s ability to reach food or water, significant tumor ulceration, guarding behavior, or upon becoming moribund. After hindlimb amputation, mice were followed with weekly bioluminescence imaging until they reached a total body bioluminescent flux of 1 x 10^10^ photons or if other endpoints were seen such as persistent poor grooming or lethargy; > 20% body weight loss post-amputation, respiratory difficulty or upon becoming moribund.

#### Tibial Implants

LM7.ffluc cells were harvested at confluence and pelleted by centrifugation. 5X collagen neutralization buffer was prepared by mixing 2.5 g minimum essential media (MEM) alpha powder without nucleosides (Thermo Fisher Scientific, Waltham, MA) and 2% wt/vol NaHCO_3_ in 45 ml demineralized water, adding 5 ml of 1 M HEPES (Thermo Fisher Scientific, Waltham, MA), and filtering through a 0.22 μm filter. Neutralized high-concentration type I rat-tail collagen was prepared fresh by mixing high-concentration rat tail collagen I (Corning, New York, USA; concentration range 8 – 11 mg/ml) and 5X collagen neutralization buffer in a 5:1 vol/vol ratio on ice. LM7 cells were then resuspended in neutralized high-concentration collagen at 1 x 10^6^ cells per 10 μL collagen, taking care to maintain reagents and pelleted cells on ice during resuspension process and pipetting using wide-bore pipette tips. Once resuspended, cell mixture was pulse-vortexed and pulse-centrifuged for < 5 seconds to disrupt bubbling in mixture. Cells were then plated for individual implants at 10 μL cell mixture per well in a 96-well ultra-low-attachment round-bottom plate (Corning, NY, USA) and allowed to solidify at 37°C in 5% CO_2_ for 20 minutes. DMEM supplemented with 10% fetal bovine serum and 1% Glutamax was then added and implants allowed to mature overnight at 37°C in 5% CO_2_. The collagen preparation and neutralization protocol described above is as that previously described (21).

#### Orthotopic Implantation

**Figure 1:**
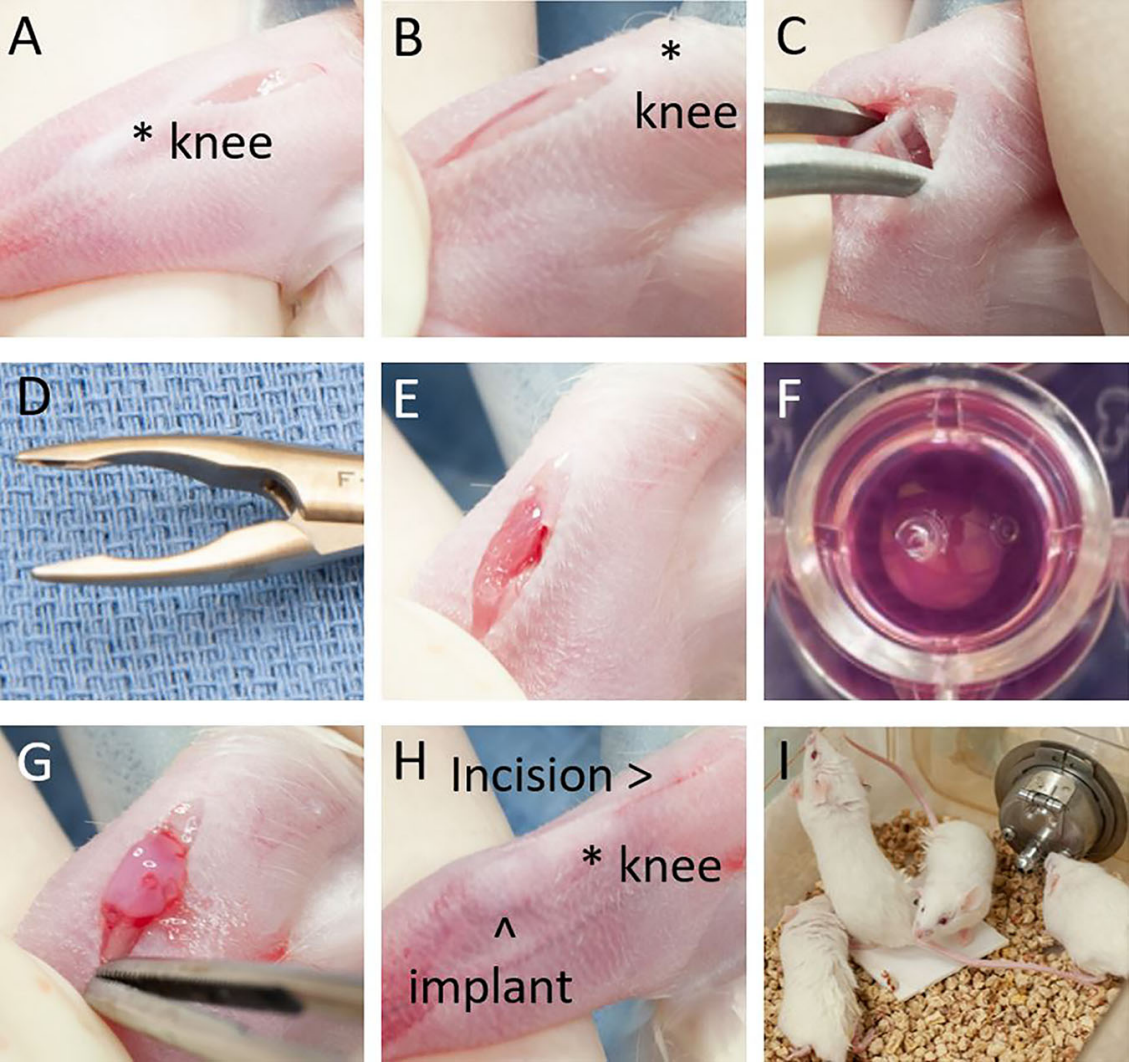
Tibial osteotomy implantation procedure. **(A)** Incision is made over distal femur. **(B)** Incision retracted distally to expose anterior tibia. **(C)** Soft tissue dissected away from anterior tibia. **(D)** 2 mm tip Rongeur used for osteotomy. **(E)** Tibial osteotomy. **(F)** LM7-collagen implant. **(G)** Implant in place over osteotomy. **(H)** Final position of implant and incision. **(I)** Mice demonstrating exploration and weight bearing behavior minutes after anesthesia emergence.

Prior to beginning the implantation procedure, mice are anesthetized using inhaled isoflurane at a MAC of 2, depilated, and the right hindlimb prepped from the inguinal area to the paw using 70% alcohol and chlorhexidine solution. Multimodal analgesia was administered both preemptively with meloxicam 5 mg/mL (Boehringer Ingelheim, St. Joseph, MO), subcutaneously at 1 mg/kg and post-operatively with buprenorphine 0.03 mg/mL (Patterson Veterinary, Greeley, CO), subcutaneously at 0.1 mg/kg. The mouse is placed in dorsal recumbency with the right hindfoot gently grasped while flexing the knee. A 5 mm skin incision is made proximal to the patella (**Figure 1A**) and retracted using gentle traction below the patella to expose the proximal anterior tibia (**Figure 1B**). The musculature and soft tissue are gently dissected away from the anterior tibia using a fine hemostat (**Figure 1C**; Fine Science Tools, Foster City, CA, USA). Once cleared of soft tissue, a 2 mm fragment of anterior tibial cortical bone is removed using a 2 mm sharp Rongeur (**Figures 1D, E**; Fine Science Tools). Care is taken to avoid the inferior patellar tendon and to avoid significant entry into the marrow cavity or tibial fracture. An LM7 collagen implant (**Figure 1F**) is then grasped with forceps and placed gently into the cavity left at the osteotomy site (**Figure 1G**). The distal aspect of the skin incision is then gently raised over the implant and the incision allowed to recoil back to its original position proximal to the patella, leaving the implant secured in place by intact skin (**Figure 1H**). Gentle pressure is applied to ensure hemostasis and implant adherence to the bony surface. The skin incision is closed with Vetbond^®^ surgical glue (3M Animal Care Products, St. Paul, MN). Mice are monitored through until completely recovered from anesthesia (**Figure 1I**), with the entire procedure from incision to closure taking 2 – 5 minutes to perform.

#### Hindlimb Amputation

Mice are anesthetized with surgical prep performed from the right hindlimb to the umbilicus. Preemptive analgesia is administered as described above and a 1 mL bolus of sterile saline is administered subcutaneously. The surgical area is draped with the hindlimb extended and secured in place with a sterile adhesive bandage.

A skin incision is made in an elliptical fashion along the inguinal canal from the rostral dorsal iliac spine to the level of the pubic ramus. Gentle blunt dissection with a cotton tipped applicator is used to push the peritoneum and abdominal musculature rostrally to expose the proximal aspect of the femoral neurovascular bundle (artery, vein, and nerve). The bundle is carefully dissected with a fine hemostat and controlled proximally and distally with 5-0 Vicryl^®^ (Ethicon Inc, Somerville, NJ) braided absorbable suture ties. Axonotmesis is performed on the femoral nerve as the bundle is divided with sharp fine scissors. The musculature overlying the femur is gently divided using sharp dissection and the femur grasped distally using toothed forceps. A heavy scissor is used to divide the femur at the mid-shaft. The distal femur is elevated, allowing visualization of the caudal musculature, which is sharply divided. Axonotmesis is performed on the sciatic nerve as it is visualized caudally using smooth forceps 3 mm proximal to the division site and then ligated. The remaining soft tissue is sharply divided, and the skin incision completed to remove the hindlimb. Care is taken to leave a sufficient posterior muscle flap to cover the proximal femur stump.

After removal of the hindlimb from the field, gentle pressure is used to ensure hemostasis. A single figure of eight suture is used to cover the proximal femur stump with the caudal muscle flap. Skin edges are adhered with an intradermal suture pattern using 5-0 Vicryl^®^ suture. Vetbond^®^ surgical glue is applied. The animal is maintained on heat throughout the surgery and during anesthetic recovery with continuous monitoring. The entire procedure from incision to closure lasts 15 – 25 minutes. Amoxicillin 400 mg/50 mls (Sandoz, Princeton, NJ) is added to a 350 ml water bottle at a dosage of 50 mg/kg for one week. Mice are monitored closely at least twice a day, in addition to regular health checks following surgery for 7-10 days by experienced veterinary technologists.

### Xenograft *In Vivo* Antitumor Model

For the B7-H3-CAR dose escalation experiment, mice underwent orthotopic LM7 implantation as described above and were monitored for engraftment and growth by weekly bioluminescent imaging. Each mouse was imaged from the ventral aspect both with and without lower extremity shielding to allow for assessment for pulmonary metastases. At day 47 post-implantation, based on bioluminescent flux of 10^8^ – 10^9^ photons/second (p/s) and visible tumor masses, mice were injected *via* tail vein with either B7-H3-CAR T cells at 3x10^5^, 1x10^6^, 3x10^6^, or 1x10^7^ T cells per mouse, or control T cells at 3 x 10^6^ per mouse. Only mice with demonstrably engrafted tumors on bioluminescent imaging were treated, leaving groups of 4 – 5 mice each. Mice were then monitored weekly using bioluminescence imaging. At reaching physical endpoints as described above, mice underwent hindlimb amputation with harvest of tumor tissue and ongoing bioluminescence imaging. Mice subsequently underwent ongoing imaging and sacrifice at physical humane endpoints or when systemic metastatic spread was consistently present in multiple organ systems and for at least 2 consecutive weeks. The right lung of each euthanized animal was reserved for dissociation and flow cytometry, and the left lung submitted for pathologic analysis.

### Xenograft *In Vivo* CAR T Cell Trafficking Model

For the *in vivo* CAR T cell trafficking assay, mice underwent orthotopic unlabeled LM7 implantation as described above. On day 28 post implantation, mice were injected *via* tail vein with 3x10^6^ B7-H3-CAR T cells or control T cells labelled with ffluc. Mice were then followed by daily bioluminescence imaging for 5 days, followed by 2 times per week for a total of 14 days.

### Bioluminescent Imaging

Mice were injected i.p. with 150 mg/kg of d-luciferin 5 minutes before imaging, anesthetized with isoflurane (1.5% - 2% delivered in 100% oxygen at 1 L/min), and imaged with an *in vivo* imaging system (IVIS 200; PerkinElmer, Waltham, MA, USA). The photons emitted from the luciferase-expressing cells were quantified using Living Image software (PerkinElmer). Mice were imaged once per week to track tumor burden, and once per day to track T cell trafficking.

### Histopathological Examination

All mice, either upon sacrifice or upon reaching the end of the study, underwent necropsy. The primary tumor site, the left lung of each mouse, and any sites of obvious extrapulmonary metastatic disease were harvested and fixed in 10% neutral-buffered formalin, embedded in paraffin, sectioned at 4 µm, and stained with hematoxylin and eosin (HE). Bony tissues were decalcified in 10% formic acid. Stained sections were visually reviewed using a Nikon Eclipse Ni upright microscope (Nikon Instruments Inc., Melville, NY) by a board-certified veterinary pathologist.

### Immunohistochemistry

All formalin-fixed, paraffin-embedded (FFPE) tissues were sectioned at 4 μm, mounted on positively charged glass slides (Superfrost Plus; Thermo Fisher Scientific, Waltham, MA), and dried at 60°C for 20 min. The following antibodies and procedures were used to detect immunohistochemical markers. CD276 (Clone RBT-B7-H3, Rabbit Monoclonal, IgG) and GFP (Clone JL8, Clontech, #632381,1:2000) were separately detected using HIER with cell conditioning media 1 (CC1, 950-224, Ventana Medical Systems, Tucson, AZ) for 32 minutes at 37°C followed by visualization with DISCOVERY OmniMap anti-Rb HRP (760-4311; Ventana Medical Systems, Tucson, AZ), and DISCOVERY ChromoMap DAB kit (760-159; Ventana Medical Systems, Tucson, AZ) or DISCOVERY ChromoMap Purple kit (760-229; Ventana Medical Systems, Tucson, AZ), respectively. Positive and negative tissue controls and isotype controls for monoclonal antibodies were used to assess the specificity of immunostaining.

### Flow Cytometric Assessment for Primary and Pulmonary Metastatic Lesions

At necropsy, half of any residual primary tumor and the right lung of each mouse was harvested and dissociated manually. Dissociated tissue was passed through a 70 μm cell strainer and washed. Cells were resuspended in PBS with 1% FBS. Flow cytometry was performed as detailed in section 2.4. Tumor cells were detected by GFP fluorescence and the percent of GFP-positive cells in each lung specimen was quantified.

### Statistical Analysis

For comparison of 3 or more groups with a single independent variable, statistical significance was determined by one-way ANOVA with a Tukey’s multiple comparison test. For comparison of three or more groups with two or more independent variables, statistical significance was determined by two-way ANOVA with Sidak’s multiple comparison test. Cumulative incidence and survival curves were plotted using the Kaplan-Meier method. Statistical significance between survival curves was determined using the long-rank (Mantel-Cox) test. For curves generated over time (cytotoxicity, bioluminescence over time), where appropriate, area under the curve was determined for each subject. Mean AUC was compared between groups using either two-tailed student’s t test for two group analyses or one-way ANOVA for three or more groups with a single independent variable.

## Results

### B7-H3-CAR T Cells Exhibit Anti-Osteosarcoma Activity *Ex Vivo*

**Figure 2:**
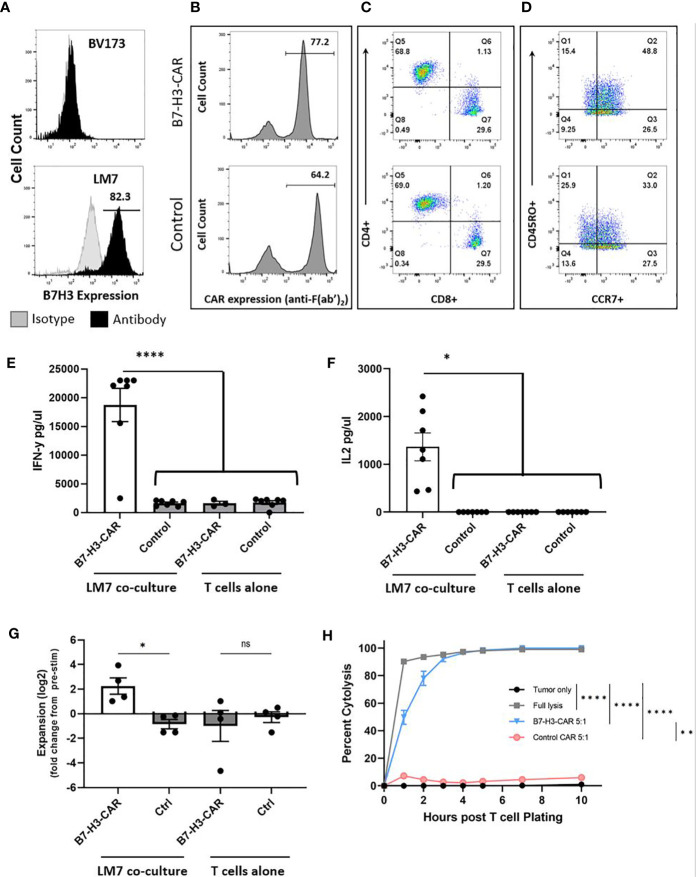
LM7 OS B7-H3 expression, CAR T cell transduction, phenotype, and *in vitro* effector function. **(A)** The LM7 OS cell line was evaluated by flow cytometry for surface B7-H3 expression and shows robust expression. BV173 cells served as known negative controls. Activated T cells were transduced with lentiviral particles encoding B7-H3.CD28ζ CARs or a control CAR (B7-H3.CD8α.Δ). B-D) Representative flow plots of transduced T cells. **(B)** Percent transduction of B7-H3-CAR and control CAR T cells. **(C)** CAR T cell CD4+/CD8+ phenotype. **(D)** CAR T cell CD45RO+/CCR7+ memory phenotypes. E-F) CAR T cells were placed in coculture with LM7 tumor cells or plated alone at a 1:1 effector:target ratio. After 24 hours, supernatant was removed and assessed by ELISA for cytokine production. B7-H3-CAR T cells demonstrated robust **(E)** IFN-γ (p < 0.0001) and **(F)** IL-2 (p < 0.05) production. N = 7 donors; performed in duplicate. Statistical analysis by one-way ANOVA. **(G)** CAR T cells were placed in coculture with LM7 tumor cells or plated alone and fold change from baseline quantified as described in the text (N = 4 donors; performed in duplicate; p < 0.05; statistical analysis by one-way ANOVA). **(H)** Impedance-based cytotoxicity assay (xCELLigence) using LM7 cells as targets demonstrated robust cytotoxicity of B7-H3-CAR T cells compared to controls (N = 4 donors; E:T ratio = 5:1; statistical analysis by AUC and one-way ANOVA; *p < 0.05; **p < 0.01; ****p < 0.0001). ns, not significant.

To establish CAR T cell activity against OS in our novel spontaneously metastasizing orthotopic model (**Figures 1A–I**), we first evaluated their function *ex vivo*. We chose to target B7-H3 in these studies because B7-H3 i) is highly expressed in a majority of OS samples (15–17), ii) is associated with OS progression/metastasis (22), and iii) has limited expression in normal human tissues (15, 16, 23, 24). Likewise, the LM7 OS cell line (25) we used to develop this model expresses high levels of B7-H3 (**Figure 2A**). We chose LM7 for this model given its development as a lung metastatic derivative of the well-characterized SaOS OS cell line (18).

B7-H3-CAR T cells or T cells expressing a nonsignaling version of the B7-H3-CAR (Control (Ctrl)-CAR T cells) were generated by lentiviral transduction as previously described (15), with resultant high level CAR expression (**Figure 2B**). Phenotyping of CAR-positive cells demonstrated comparable CD4:CD8 ratios and a predominance of T cells with a memory phenotype (CD45RO+/CCR7+ or CD45RO+/CCR7-) (**Figures 2C, D**). To evaluate their effector function, CAR T cells were incubated with LM7 tumor cells and supernatant was collected 24 hours later to measure cytokine production. B7-H3-CAR T cells secreted significantly greater IFNγ and IL-2 compared to Ctrl CAR T cells (**Figures 2E, F**; N = 7 donors; p < 0.0001 for IFNγ; p < 0.05 for IL-2). No significant cytokine production was observed by T cells in the absence of tumor cells, confirming that cytokine production occurred due to CAR recognition of the tumor cells. B7-H3-CAR T cells also expanded in the presence of LM7 cells in contrast to Ctrl-CAR T cells (**Figure 2G**; N= 4-5 donors; p < 0.05). This expansion was antigen specific since no significant difference was observed in the absence of tumor cells between B7-H3- and Ctrl-CAR T cell populations. -CAR T cell cytotoxicity was measured using an xCELLigence impedance-based assay. B7-H3-CAR T cells rapidly killed LM7 OS cells, reaching > 95% cytolysis 5 hours post co-culture (**Figure 2H**). In contrast, Ctrl CAR T cells exhibited minimal antitumor activity (N = 4 donors, statistical analysis by AUC and one-way ANOVA; *p < 0.05; **p < 0.01; ****p < 0.0001).

### B7-H3-CAR T Cells Exhibit Dose-Dependent Antitumor Activity in the Established Orthotopic Spontaneously Metastasizing Xenograft Model

Twenty-five 8-week old NSG mice were implanted with collagen-embedded LM7.GFP.ffluc in the anterior tibial crest according to the procedure described in the Material and Methods section and shown in **Figures 1A–I**. All mice survived general anesthesia and were weight-bearing, grooming, and had normal cage exploration behaviors within 10 minutes of anesthetic recovery. There were no perioperative complications such as wound dehiscence, infection, bleeding, intractable pain, or evidence of osteomyelitis. A perioperative analgesic regimen of daily meloxicam and buprenorphine for 5 – 7 days resulted in satisfactory pain control after tibial osteotomy. Out of 25 implanted mice, 22 (88%) developed robust tibial tumors detectable by bioluminescent imaging and visual inspection and were used for further studies.

**Figure 3:**
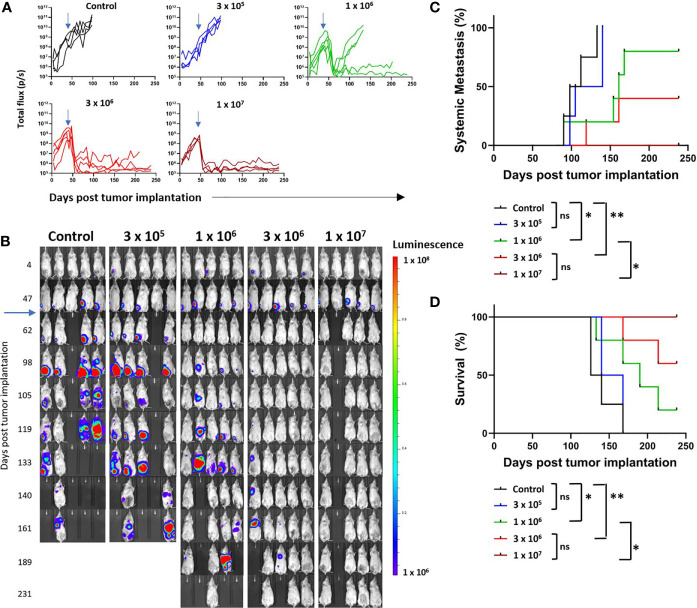
Growth and metastasis of LM7 OS cells in orthotopic model. LM7 OS cells expressing firefly luciferase were embedded in highly-viscous collagen and implanted into the tibia of NSG mice on Day 0. On day 48, CAR T cells were injected by tail vein at indicated doses (blue arrows). Mice were followed by serial bioluminescence imaging and underwent hindlimb amputation or sacrifice as described in the text. **(A)** Quantified growth of OS tumors after implantation (total flux (p/s) per ROI). **(B)** Serial imaging of implanted mice over time. **(C)** Cumulative incidence curve indicating development of systemic disease as determined by non-tibial bioluminescent signal appearance in two consecutive images. **(D)** Kaplan-Meier curve indicating overall survival of mice. N = 4-5 mice/group, 1 healthy T cell donor. Statistical analysis performed by log-rank testing. *p < 0.05; **p < 0.01. ns, not significant.

Four escalating doses of B7-H3-CAR T cells derived from a single healthy donor were injected by tail vein (iv) (3x10^5^, 1x10^6^, 3x10^6^, or 1x10^7^ cells/mouse) 48 days post tumor implantation. In addition, Ctrl-CAR T cells were injected iv at 3x10^6^ cells per mouse. Post injection, B7-H3-CAR T cells exhibited antitumor activity in a dose-dependent manner (**Figures 3A, B**). All Ctrl- and low-dose B7-H3-CAR (3x10^5^) T cell treated mice had progressive primary disease and required hindlimb amputation by day 100. Overall, 14 mice achieved baseline primary tumor control with CAR T cell therapy. All mice in the intermediate (1x10^6^), intermediate-high (3x10^6^), and high (1x10^7^) dose groups initially had complete primary tumor response to CAR T cell treatment (**Figures 3A, B**). However, 2 mice in the intermediate dose group recurred at the primary site and required amputation at day 135. At intermediate-high and high doses, primary tumors responded completely to B7-H3-CAR T cell treatment and none required amputation, demonstrating robust CAR T cell antitumor activity (**Figures 3A, B**). At study completion, mice in the intermediate-high and high dose treatment groups were euthanized and pathologic examination of the tibial implantation sites revealed no tumor cells, confirming the complete responses determined by bioluminescence imaging.

As one of the major goals of this study was to evaluate systemic metastatic disease, amputation was performed to enable survival, and not to prevent disease spread. Development of imaging-defined systemic metastasis was defined as two consecutive weeks of extra-tibial bioluminescent signal above flux of 1 x 10^6^ p/s. Our orthotopic implantation method resulted in a high propensity for spontaneous OS metastasis. All Ctrl- and low dose B7-H3-CAR T cell treated mice developed systemic metastatic disease despite hind limb amputation (**Figures 3B, C**). Notably, two mice in the control group had evidence of metastatic disease prior to hindlimb amputation performed on day 100 (**Figure 3B**). At the intermediate dose two mice developed metastatic disease despite showing a complete response at the primary tumor site without amputation (**Figures 3B, C**), and 2 mice in the intermediate-high dose group developed metastatic disease despite B7-H3-CAR T cell control of the primary tumor. No mice in the high dose treatment group developed systemic metastasis detectable by bioluminescent imaging. At 6 months, there was a clear and statistically significant dose-dependent difference in metastasis development (**Figure 3C**) and survival advantage for B7-H3-CAR T cell treated mice (**Figure 3D**). In total, 8 mice remained long-term survivors: 1/5 in the intermediate dose group, 3/5 in the intermediate-high dose group, and 4/4 in the high dose group.

These data demonstrate utility of this model for evaluating CAR T cell activity and demonstrate that B7-H3-CAR T cells control local OS and prevent metastatic disease in a dose-dependent fashion.

### Established Orthotopic Spontaneously Metastasizing Xenograft Model Allows Non-Invasive Monitoring of B7-H3-CAR T Cell Trafficking to Tumors

**Figure 4:**
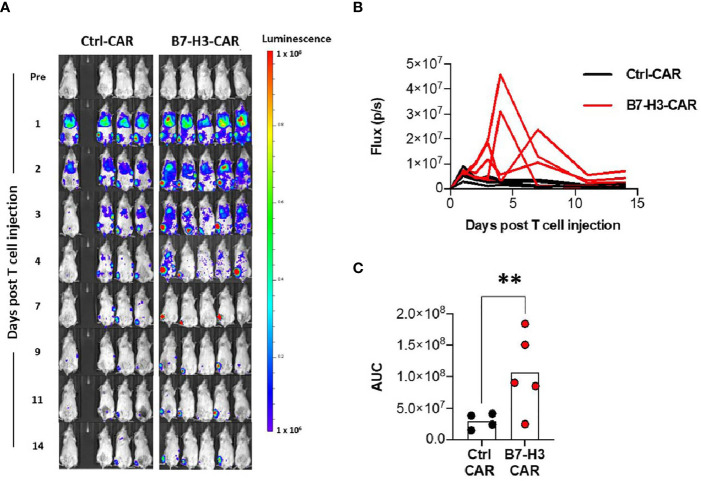
CAR T cell trafficking to orthotopic tumors. LM7 OS cells were embedded in highly-viscous collagen implanted into the tibia of NSG mice on Day 0. On day 28, B7-H3-CAR T cells expressing firefly luciferase were injected *via* tail vein. Mice were then imaged with serial bioluminescence over the following 14 days. **(A)** Serial imaging of implanted mice over time. **(B)** Quantification of tibial implant site bioluminescence (total flux (p/s) per ROI) over time. **(C)** AUC calculation for B7-H3-CAR (N = 5) and Ctrl-CAR (N = 4) treated mice. One healthy T cell donor. Statistical analysis performed by student’s t test. **p < 0.01.

We next explored if the orthotopic implant OS model could be used to monitor T cell trafficking and expansion at the primary tumor site. Ten mice were implanted with collagen-embedded LM7 cells, and twenty-eight days later, 3x10^6^ ffluc-expressing B7-H3- or Ctrl-CAR T cells were injected *via* tail vein into 9 surviving mice (one mouse unexpectedly died before treatment). Bioluminescent imaging was performed daily for 5 days, followed by 2 times per week to track T cells *in vivo*. After exiting the pulmonary vasculature, B7-H3-CAR T cells trafficked to engrafted right tibial tumors beginning on day 3 post implantation and exhibited significantly (**Figures 4A–C**; p < 0.01) greater tibial expansion on day 4 post-injection compared to Ctrl-CAR T cells. In addition, B7-H3-CAR T cells persisted at the primary tumor site through 14 days post-injection. In contrast, Ctrl-CAR T cells, while exhibiting similar early trafficking to right tibial tumors, did not expand and had minimal persistence beyond 7 days post-injection (**Figures 4A, B**).

### Orthotopic LM7 Tumor Implantation Produces Robust Primary and Metastatic Disease and Tumors Have Essential Characteristics of OS

**Figure 5:**
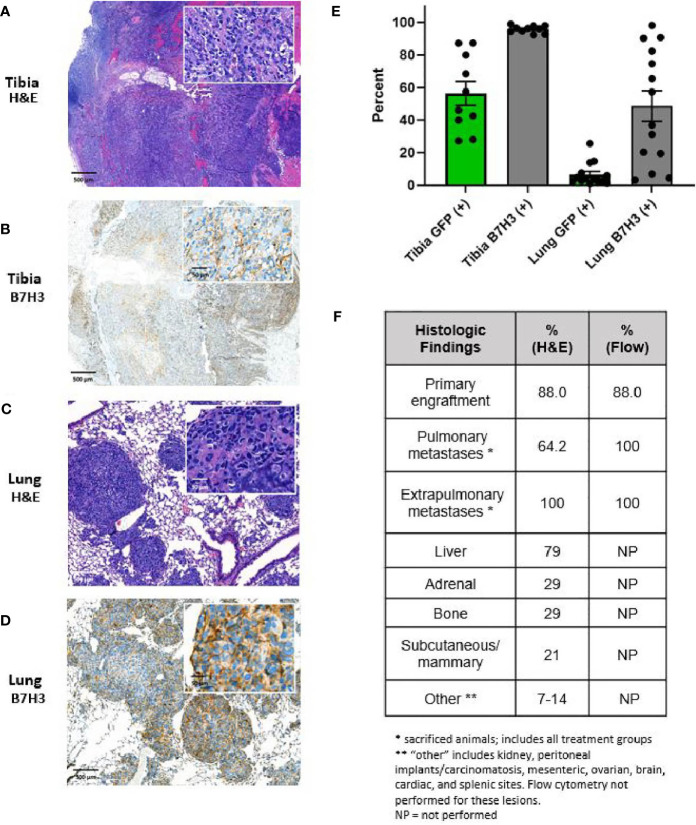
Characterization of primary and metastatic lesions. Primary tibial osteosarcoma lesions are characterized by **(A)** H&E and B) B7H3 immunohistochemical staining, demonstrating formation of lesions with the appearance of malignant osteoid and invasion of the cortex and marrow cavity as well as B7H3 expression. Pulmonary metastases are characterized by **(C)** H&E and **(D)** B7-H3 immunohistochemical staining, demonstrating consistency of metastatic lesions with osteosarcoma lesions and persistent B7-H3 after metastasis. For **(A–D)**, scale indicated is 500 μm for low power images and 50 μm for high power inserts. **(E)** Percent of tumor cells (GFP+) and B7-H3 expression on tumor cells (B7-H3+) as assessed by flow cytometry. Primary tumor sites contained high percentages of human tumor cells as assessed by GFP fluorescence (mean 56.5%, SE 7.3%). Tumor cells were additionally detected in all right lungs of mice not achieving long-term survival (mean 6.6%, SE 1.9%). B7-H3 expression was additionally demonstrated in all primary lesions (mean 95.8%, SE 2.2%) and pulmonary metastatic lesions (mean 48.7%, SE 9.4%). **(F)** Percent of animals with primary site engraftment, pulmonary metastases, and other distant hematogenous metastases on post-mortem histology and/or post-mortem flow cytometry (as applicable) on recovered tissue.

All mice in the dose-escalation CAR T cell treatment experiment underwent tissue harvest at hindlimb amputation, and necropsy was performed at terminal endpoints to allow pathologic evaluation of tumor and lung tissues. Extrapulmonary sites of metastasis were quantified at necropsy and selectively sampled for further studies. Engrafted tumors demonstrated cortical disruption and intramedullary extension of tumor burden. **Figure 5A** demonstrates a representative coronal section through the proximal tibia, showing both the distal femur and proximal tibia, the associated joint space, and surrounding soft tissue. Engrafted OS disrupted the cortical surface, extends into the medullary cavity, and established on both the anterior surface of the tibia and extended posteriorly into the soft tissue of the thigh and calf. All engrafted primary tumors exhibited pleomorphic spindle-shaped cells with a high mitotic index and nuclear pleomorphism. In addition, deposition of malignant osteoid was observed, consistent with OS (**Figure 5A**). Additionally, all engrafted tumors exhibited strong B7-H3 immunostaining, consistent with expression of B7-H3 in the LM7 cell line (**Figure 5B**).

Of the 8 mice in the control and low dose treatment groups, 6 had evidence of pulmonary metastasis on lung H&E sections. Five of these exhibited multifocal nodular metastases, and 1 in the low dose group exhibited multifocal neoplastic emboli without established metastasis. Of the 6 mice in the intermediate- and intermediate-high treatment groups that were euthanized due to progression of systemic metastasis, 4 showed evidence of micro-metastasis on H&E. Nodular lung metastases exhibited similar characteristics to the primary tumor site, with high nuclear pleomorphism, high mitotic rates, and malignant osteoid deposition (**Figure 5C**). Pulmonary lesions also stained positive for B7-H3 (**Figure 5D**). Primary and pulmonary specimens were additionally assessed for surface B7-H3 expression and evidence of metastatic spread to the lungs by evaluating GFP and B7-H3 positive cells within the primary tumor and pulmonary tissue. Fragments of primary tumor tissue and the right lung of each mouse were made into single-cell suspension and evaluated by flow cytometry for B7-H3 and GFP expression (**Figure 5E**). GFP expression was detected in all persistent primary tumors and in all pulmonary specimens from mice that did not achieve long-term survival, indicating presence of GFP-positive tumor cell spread to the lungs in these mice even in cases where metastasis was not demonstrated by H&E. Additionally, all primary and metastatic tumors exhibited ongoing B7-H3 expression (**Figure 5E**). Finally, extrapulmonary metastatic spread was noted in all mice examined by H&E. The primary extrapulmonary metastatic sites included liver, adrenal glands, kidney, axial bony sites, mesenteric and serosal surfaces including presence of carcinomatosis, and others (**Figure 5F**).

## Discussion

In this study, we describe a novel spontaneously metastasizing orthotopic model of OS with several advantages over existing methodologies. These include i) a high rate of spontaneous pulmonary and extrapulmonary systemic metastases, ii) lack of immediate pulmonary seeding *via* marrow injection, iii) easily accessible site for implantation and subsequent primary tumor amputation, and iv) ability to use modified or suspension cellular material, such as ffluc-modified tumor cells for noninvasive bioluminescent imaging, without resorting to intramedullary injection. The model resulted in primary tumor engraftment in 88% of mice in this study, with 58% and 100% of CAR T cell non-responders developing pulmonary metastasis by H&E and flow cytometry respectively, and 100% of non-responders developing extrapulmonary metastatic disease. Considering these advantages and the high rate of systemic metastasis, this model fills a gap in the currently available methodology for studying OS in orthotopic and metastatic settings. A comparison of our model with currently available orthotopic models is summarized in **Table 1** and discussed in further detail below.

**Table 1:** Comparison of murine orthotopic osteosarcoma models.

Model	Characteristics						
	Modified or selected cells	Whole tissue fragments	Inoculum spread controlled	Direct pulm seeding	Spont pulm mets	Ease of access	
						Implantation & imaging	Amputation
**This study**							
Tibial osteotomy with collagen implant or fragment transplantation	Y	Y	Y	N	Y	Y	Y
**Previous studies**							
Periosteal activation with cell injection or fragment transplantation (26)	Y	Y	N	N	Y	Y*	Y*
Intraosseous cell injection (27)	Y	N	Y	Y	N	Y*	Y*
Femoral fragment transplantation (28)	N	Y	Y	N	Y	N	N

Spont, spontaneous; pulm, pulmonary; mets, metastasis; *, tibial site only.

Using our model, we demonstrated here that B7-H3-CAR T cells exhibit antitumor activity against primary and metastatic OS in a dose-dependent fashion. In mice treated with intermediate-high and high CAR T cell doses, we have achieved complete response and long-term survival of > 6 months from treatment. We have shown that different CAR T cell doses result in different patterns of primary tumor response and subsequent metastasis development in a way that mimics clinical surveillance of human patients and their outcomes.

Despite considerable preclinical promise for CAR T cell therapies in sarcomas, including osteosarcoma, rhabdomyosarcoma, synovial sarcoma, and Ewing sarcoma, clinical efficacy has remained elusive. A single phase I/II clinical trial for pediatric sarcoma has been reported (12), although several are now actively recruiting (NCT00902044, NCT02107963, NCT01953900, NCT03635632, NCT04483778, NCT03618381), and clinical trials have been reported for pediatric patients with glioblastoma (29) and neuroblastoma (30, 31). However, these clinical trials have not demonstrated robust antitumor activity in humans. The failure in clinical trials has been due to either lack of response, with patients exhibiting disease progression after CAR T cell therapy, or partial responses that progress after treatment. Very few complete responses have been noted. These failures raise concerns that preclinical models available for pediatric sarcomas, specifically OS, are not of sufficiently high-fidelity to allow adequate preclinical vetting of antitumor efficacy. Preclinical modeling for CAR T cell therapy in bone tumors, including OS and Ewing sarcoma, has relied on several strategies to assess *in vivo* efficacy. For primary tumor modeling, these have included periosteal injection (26, 32), intratibial injection (11, 33–35), an intraperitoneal loco-regional model (13, 36), and subcutaneous injection (34, 37–41). Other investigators have used an orthotopic OS model to evaluate T cells expressing an MGA271 scFv-based B7-H3-CAR with a 41BB costimulatory domain (26). Based on our previous publication, which demonstrated improved antitumor activity of MGA271 scFv-based B7-H3-CAR with a CD28 versus a 41BB costimulatory domain (15), we selected a CAR with a CD28 costimulatory domain for this study. Clearly, future studies should focus on directly comparing MGA271 scFv-based B7-H3-CARs with different costimulatory domains, including CD28, 41BB, and others, in this model to fully understand the different tumor control capacities of these CARs.

We performed a half-log dose de-escalation study in our model evaluating a dose range of 1 x 10^7^to 3 x 10^5^ CAR T cells per mouse. We chose a starting cell dose of 1 x 10^7^CAR T cells because 1 – 2 x 10^7^ CAR T cells per mouse is a routinely accepted maximum cell dose in preclinical CAR T cell xenograft models. For the Ctrl CAR T cell group, we evaluated a cell dose of 3 x 10^6^ CAR T cells per mouse, which approximated our maximum B7-H3-CAR T cell dose. Based on our results at this cell dose (no antitumor activity of Ctrl-CAR T cells; excellent antitumor activity with long-term survival of > 100 days post infusion of B7-H3-CAR T cells), we felt that it was not justified to perform additional animal experiments at lower Ctrl-CAR T cell doses.

The most common orthotopic method of OS inoculation involves intratibial injection of single cell suspensions using a heavy gauge needle (27). This model system has been extensively used for chemotherapeutic investigation, metabolic research, and immunotherapy in osteosarcoma in primary and metastatic settings (11, 42–52). A similar protocol has been described for intra-femoral injection (53–55). This injection method results in robust primary orthotopic tumor engraftment, and indeed, has been used to demonstrate antitumor activity of CAR T cells against bone tumors (11, 14, 33, 35, 37). However, direct injection of OS cells into murine femurs or tibias has been shown to result in direct pulmonary seeding due to venous outflow from the medullary cavity, making it suboptimal as a model of osteosarcoma metastasis (56, 57). While this may be avoided by using extremely low cell inoculums or attempting low-pressure injection (58), such strategies may not be feasible for all model systems or reliably reproducible between technicians. Our method avoids this problem while maintaining robust orthotopic engraftment by confining the tumor cells within a collagen scaffold, thereby avoiding direct medullary injection and venous dissemination while providing a high rate of spontaneous metastasis.

Two groups have reported using a periosteal injection strategy to establish primary orthotopic OS tumors without intramedullary injection for assessing CAR T cell therapy. In the first, periosteal injection of OS cells resulted in successful engraftment of primary OS tumors with rapid growth (26). The second group utilized the highly aggressive 143B OS cell line in a periosteal injection method to assess primary tumor growth (32). This method produces cortical OS and does not result in immediate pulmonary seeding. It can also be applied to both implantable fragments and single cell suspensions, provided the injected cells remain in apposition to the scored periosteum. However, this method can result in soft tissue or periosteal inoculation as a result of imprecise localization of the injected cells, thereby not reliably achieving cortical and intramedullary OS engraftment (59, 60). Finally, the metastatic rate of models using this method is relatively low, and the injection of single cell suspensions into the paracortical position can result in less control over the spread of the suspension into the soft tissue than placement of a tumor fragment or implant as we have described here (60–62). Subcutaneous and intraperitoneal locoregional models of OS, which are the other major methodologies employed in murine CAR T cell preclinical models, do not recapitulate the microenvironment of skeletal OS and are known to have inferior metastasis rates compared to orthotopic implantation (63).

Metastatic OS models for CAR T cell evaluation have included IV injection of OS cells (35, 36, 40), resulting in immediate seeding but allowing for evaluation of stabilization and growth in the pulmonary niche, and in two cases assessment of either number of pulmonary metastases at endpoint by histology or overall survival after hindlimb amputation (14, 26), which is imputed to treatment of metastatic sites. While IV injection of tumor cells results in reliable pulmonary OS seeding, it does not allow for high-fidelity recapitulation of the metastatic process, as it avoids the steps of primary tumor escape, vascular entry, and metastatic site extravasation.

An additional option for orthotopic investigation of OS, not yet described in CAR T cell preclinical studies, involves surgical exposure of the distal femur of the mouse, resection of the lateral femoral condyle, and implantation of a fragment of fresh tumor tissue (28). This method has the advantage of providing an orthotopic implantation site without injection of tumor cells and allows implantation of fresh fragments of tumor, which is valuable for applications requiring intact tumor-stroma connections. It also does not result in direct seeding of the pulmonary metastatic site. However, it does not provide a method for orthotopic implantation of single cell suspensions, and therefore is limited in terms of the ability to use bioluminescence or fluorescent noninvasive monitoring of primary tumor growth without first establishing xenograft donor tumors (i.e., by subcutaneous injection and subsequent harvest). In addition, the site itself is buried within the lateral musculature of the animal’s hindlimb, making direct visual monitoring of the tumor site difficult and hindlimb amputation for long-term tumor studies technically challenging. The presence of proximal tumor and/or intramedullary extension throughout the femoral medullary canal means that amputation must be performed near or through the acetabular/glenoid junction, leading to technical difficulty related to adequate dissection of the joint capsule and avoidance of the femoral artery and vein at an anatomic site of close apposition to the joint capsule. These technical difficulties may limit the performance of hindlimb amputation to more highly trained technicians or researchers.

Two groups of investigators have described intratibial implantation of histologically intact fresh tumor tissue into the tibia of mice (64, 65). Both models closely resemble that described in this report, but do not allow the use of modified cells or single cell suspensions for noninvasive tumor growth and imaging applications. They also do not allow the use of specialized cell line variants, such as serially passaged lines with high metastatic capability (66) or gene-edited cell lines. Our model addresses this problem by additionally incorporating embedding of the tumor cells within a collagen scaffold.

This model is technically feasible. The mice used in this methodology do well after implantation. Mice are recovered from anesthesia, weight-bearing, and exploring surroundings within five minutes of awakening from general anesthesia. There is no evidence that pretreatment with buprenorphine and meloxicam affects the tumor growth in this model (67). The method is technically straightforward and easily teachable for nonsurgical personnel, and the subsequent hindlimb amputations are rendered significantly easier by use of a tibial rather than a femoral implantation site. These considerations increase the feasibility of use of this model.

Limitations of this method may include the need to acquire specialized surgical tools including the fine Rongeur used for creating the tibial osteotomy and the need to train technicians in the surgical technique. In addition, the impact of the collagen scaffold on tumor cells has not been established. We do not anticipate the collagen matrix to significantly change tumor biology and have demonstrated that this technique results in histology consistent with osteosarcoma. In addition, the use of scaffolding material such as Matrigel^®^ (Corning, Arizona, USA) is extremely common in tumor cell injection and implantation techniques, and therefore this strategy is not overly divergent from common practice. Finally, while the pulmonary metastatic potential of this model is a major advantage, the frequency and burden of the extrapulmonary systemic metastatic sites differs from the pattern of osteosarcoma metastasis in humans (68, 69). This may be due to different tumor tropism for osteosarcoma in murine systems, and indeed, extrapulmonary metastases have been reported in previous orthotopic models (61). While this metastatic pattern does differ from the clinical pattern in humans, it still allows rigorous evaluation of the metastatic process.

In summary, the orthotopic implantation technique detailed in this study, in which tumor cells are first embedded into a collagen scaffold implant and then implanted surgically into the anterior tibia of mice, results in robust primary tumor engraftment and systemic metastasis as determined by H&E and flow cytometry. We additionally describe effective antitumor activity of B7-H3-CAR T cell therapy in a dose dependent fashion using this model and show its efficacy in distinguishing primary tumor control from subsequent systematic metastasis. Thus, our model is a valuable addition to the field and should enable the realistic modeling not only of cell therapies but other therapeutics for primary and metastatic OS.

## Data Availability Statement

The raw data supporting the conclusions of this article will be made available by the authors, without undue reservation.

## Ethics Statement

The animal study was reviewed and approved by St. Jude Children’s Research Hospital Institutional Animal Care and Use Committee.

## Author Contributions

Conception and design: LT, SG, CD. Development of surgical orthotopic implantation model: LT, AD, AF. Development of nonsurgical methodology: LT, AC, PN, JW. Acquisition of data: LT, AC, PN, JW, HT. Analysis and interpretation of data: LT, AC, HT, AR, SG, CD. Writing, review, and revision of manuscript: all authors. Study supervision: AD, SG, CD. All authors contributed to the article and approved the submitted version.

## Funding

This work was supported by the Assisi Foundation of Memphis, the American Lebanese Syrian Associated Charities, and the Rally Foundation for Childhood Cancer Research Young Investigator grant program (grant 20YIN44). Animal imaging was performed by the St. Jude Center for In Vivo Imaging and Therapeutics, which is supported in part by NIH grants P01CA096832 and R50CA211481. The content is solely the responsibility of the authors and does not necessarily represent the official views of the National Institutes of Health.

## Disclaimer

The content is solely the responsibility of the authors and does not necessarily represent the official views of the National Institutes of Health.

## Conflict of Interest

SG and CD have patent and patent applications in the field of cell and gene therapy for cancer, including a patent application for B7-H3-CAR T cells. SG consults for Catamaran Bio, Nektar Therapeutics, TESSA Therapeutics, is on the Scientific Advisory Board of Tidal, and is a DSMB member of Immatics.

The remaining authors declare that the research was conducted in the absence of any commercial or financial relationships that could be construed as a potential conflict of interest.
